# Underwater Wireless Sensor Networks: How Do Acoustic Propagation Models Impact the Performance of Higher-Level Protocols?

**DOI:** 10.3390/s120201312

**Published:** 2012-01-31

**Authors:** Jesús Llor, Manuel P. Malumbres

**Affiliations:** Physics and Computer Engineering Department, Miguel Hernandez University, Ave. Universidad S/N, Ed. Alcudia, 03202 Elche (Alicante), Spain; E-Mail: mels@umh.es

**Keywords:** underwater wireless sensor networks, network simulation, acoustic propagation models, MAC and routing protocols

## Abstract

Several Medium Access Control (MAC) and routing protocols have been developed in the last years for Underwater Wireless Sensor Networks (UWSNs). One of the main difficulties to compare and validate the performance of different proposals is the lack of a common standard to model the acoustic propagation in the underwater environment. In this paper we analyze the evolution of underwater acoustic prediction models from a simple approach to more detailed and accurate models. Then, different high layer network protocols are tested with different acoustic propagation models in order to determine the influence of environmental parameters on the obtained results. After several experiments, we can conclude that higher-level protocols are sensitive to both: (a) physical layer parameters related to the network scenario and (b) the acoustic propagation model. Conditions like ocean surface activity, scenario location, bathymetry or floor sediment composition, may change the signal propagation behavior. So, when designing network architectures for UWSNs, the role of the physical layer should be seriously taken into account in order to assert that the obtained simulation results will be close to the ones obtained in real network scenarios.

## 1. Introduction

There has been an increasing interest in the development of Underwater Wireless Sensor Networks (UWSNs) in the last years. The first attempts to analyze UWSN behavior were based on the mature technology developed during the last decade in terrestrial wireless sensor networks (TWSNs). Despite having a very similar functionality, UWSNs exhibit several architectural differences with respect to the terrestrial ones, which are mainly due to the transmission medium characteristics (sea water) and the signal employed to transmit data (acoustic ultrasound signals) [1]. Then, the design of appropriate network architecture for UWSNs is seriously complicated by the conditions of the communication system and, as a consequence, what is valid for terrestrial WSNs is perhaps not valid for UWSNs, so a general review of the overall network architecture is required in order to supply an appropriate network service for the demanding applications in such an unfriendly submarine communication environment.

Basically, an UWSN is formed by the cooperation among several network nodes (often called sensor nodes) that establish and maintain the network through the use of bidirectional acoustic links. Every node is able to send/receive messages from/to other nodes in the network, and also to forward messages to remote destinations in case of multi-hop networks. Every node may have one or several sensors that are actively recording environmental data which should be forwarded to special sink nodes, typically platforms or buoys at the surface. Sink nodes have communication channels to forward (and/or local store) the collected data to the remote control station in the coast, typically through a Radio Frequency (RF) link.

Since acoustic signals are mainly used in UWSNs, it is necessary to take into account the main aspects involved in the propagation of acoustic signals in underwater environments, including: (a) the propagation speed of sound underwater is around 1,500 m/s (five orders of magnitude slower than the speed of light), and so the communication links will suffer from large and variable propagation delays and relatively large motion-induced Doppler effects; (b) phase and magnitude fluctuations lead to higher bit error rates compared with radio channels’ behaviour, being mandatory the use of forward error correction codes (FEC); (c) as frequency increases, the attenuation observed in the acoustic channel also increases, being this a serious bandwidth constraint; (d) multipath interference in underwater acoustic communications is severe due mainly to the surface waves or vessel activity, being a serious problem to attain good bandwidth efficiency.

So, designing an efficient and reliable communication channel is not an easy task, being roughly different from TWSN approaches. This fact may be the reason for the existence of a lot of simulation tools that define different models of underwater acoustic signal propagation. In fact, there is no agreement about when to use a particular simulation tool and/or standard model to represent the underwater acoustic propagation behavior; indeed there are almost as many simulation tools for this purpose as MAC and routing protocol proposals. In general, these studies have mainly been focused in developing higher layer protocols without paying much attention to the physical layer and its components. AUVNetSim [2] is an example where the physical layer is too simple, based on Thorp approximations, so different environment conditions cannot be considered in the propagation model, leading to simulation results that may be far from the ones obtained in real network deployments.

Other approaches define complex underwater acoustic propagation models that are closer to the real behavior of underwater acoustics. This is the case of the acoustic propagation model proposed by Xie and Gibson [3] which it is based on the Monterrey Miami Parabolic Equation (MMPE). The MMPE equation calculates the evolution of sound pressures produced by an acoustic source in a specific underwater scenario. It divides the scenario in a grid of 3-D cells, performing the requiring computations to get a representative sound pressure for each cell. If we reduce the cell size, we can obtain more accurate prediction results, but the computational demand for the corresponding calculations is overwhelming at medium to large size network scenarios. The MMPE model is implemented with OPNET Modeler [4], so small scale network simulations may be performed, but there is an intrinsic scalability problem when performing network simulations.

Another underwater network simulation framework is the World Ocean Simulator System (WOSS) [5]. It is composed by several tools like (a) ns2 simulator [6], (b) Bellhop tool that accurately models the underwater sound propagation by an specific ray tracing algorithm, and (3) network scenarios defined with real data (temperature, salinity, wave activity, *etc*.) from well-known world ocean databases. The WOSS framework and the MMPE model are two approaches that perform an accurate acoustic propagation modeling, but they suffer from high complexity limiting the simulation to small network scenarios and low numbers of network nodes.

In order to alleviate the complexity constraint, in [7] we proposed a statistical prediction model based on the Bellhop ray tracing tool that reduces its complexity and achieves similar levels of prediction accuracy, so we will be able to perform network modeling with reasonable high accuracy level and low computational overhead.

These modeling tools and a lot of variations around them lead to the hard task of comparing two different proposals unless they are implemented on the same platform, and even in this case, the simulator should be as realistic as possible towards the real environment conditions. Otherwise the results will lack of accuracy, and empirical testing, at least in scale-down experiments, should be done before releasing the final implementation of the underwater nodes, reducing the power of simulation tools for predicting real network behavior.

At simulation time, when we define the parameters of a network scenario and the location where network nodes would be deployed, we may use a simple assumption through general scenario parameters or define those scenario parameters that will have a direct influence in the acoustic propagation behavior. For example, we may decide to use a simple scenario where the sound speed propagation is considered as a fixed value of 1,500 m/s, with a two dimensional deployment area (depth is not considered) and a simple acoustic propagation approach like the one proposed by Thorp [8], to evaluate the performance of a point-to-point link between two nodes. On the contrary, we could define a more detailed network scenario by including, among others, the scenario location with bathymetry and floor sediment composition that will impact on the way sound propagation is reflected/absorbed at the ocean floor. Also, the temperature of the water will depend on both the latitude and longitude of network scenario and the season of the year. This fact together with the water salinity and the depth may change the sound speed between 1,450 and 1,540 m/s. There are other important factors that may change sound propagation behavior such as the well-known ocean wave’s influence which is different for shallow and deep waters, or the noise produced by ships, biological activity or shoals. All of these scenario parameters should be taken into account in order to develop detailed acoustic propagation models for UWSN, so modeling higher-level network protocols will be aware of network scenario conditions, and the obtained simulation results would be closer to the ones obtained in experimental tests [9].

In this work, we will review several acoustic propagation models from simple approaches to the more accurate ones, and observe their behavior when different network scenario parameters are changed (*i.e*., wave activity), so we can determine their sensibility to environmental network scenario parameters. Then, we will choose the most appropriate acoustic propagation model in terms of accuracy and low-complexity, in order to analyze the performance behavior of different MAC protocols and also check how the scenario environmental changes impact on their network performance in terms of throughput, delay and collisions. From the result obtained in this study, we will appreciate: (a) the importance of defining an accurate and low complexity propagation model, and (b) the sensibility of higher layer protocols to the time-varying scenario environmental conditions.

This paper is organized as follows: in Section 2, we present several acoustic propagation models, and in Section 3 we introduce the higher-layer protocols (MAC and routing protocols) used in this work. In Section 4, we evaluate the selected acoustic propagation models with a fixed MAC protocol in order to observe their impact on network performance under different scenario conditions. Then, in Section 5, we will analyze the behavior of several MAC and routing proposals under a simple and accurate acoustic propagation model. Finally in Section 6, some conclusions and are drawn future work proposed.

## 2. Acoustic Propagation Models

Simulating UWSN communications requires modeling the acoustic wave’s propagation while a node tries to transmit data to another one. There are several models proposed in the literature from the simplest ones based in the sound propagation theory to more elaborate and complex models based on the physics of acoustic sound propagation. In this section, we will describe several acoustic propagation models that represent different approaches to the same problem, but with different degrees of complexity/accuracy. We will present them in order of increasing complexity, so for each approach we will know how propagation acoustics are predicted and what parameters are taken into account for that purpose.

### 2.1. Urick Description and Thorp Formula

The theory of the sound propagation is according to the description by Urick [10], a regular molecular movement in an elastic substance that propagates to adjacent particles. A sound wave can be considered as the mechanical energy that is transmitted by the source from particle to particle, being propagated through the ocean at the sound speed. The empirical formula presented by Thorp [8] is defined as the sound intensity decrease through the path between the source and destination nodes. The absorption coefficient factor α depends on the sound frequency *f*. The proposed acoustic attenuation expression is represented as follows:

(1) $$A(d,\;f)=d^{k}\;\alpha {\left(f\right)}^{d}$$

where *d*: Distance, *k*: Geometry *(k = 1*: Cylindrical, *k = 2:* Spherical).

In the same set of formulas is also available the definition for power spectral density to calculate the noise in the receiver nodes (see [8] for more details).

### 2.2. Monterrey Miami Parabolic Equation (MMPE)

The Monterey-Miami Parabolic Equation model [11] is used to predict underwater acoustic propagation using a parabolic equation which is closer to the Helmholtz equation (wave equation) [12]; this equation is based on Fourier analysis. The sound pressure is calculated in small incremental changes in range and depth, forming a grid. It incorporates randomness and wave motion to the approximation, using a dynamic propagation loss calculation. The authors show that small changes in depth and node distances can drive to big differences in the path loss as a result of the ocean wave’s motion impact on acoustic propagation (more details in [3]). The propagation loss formula based on the MMPE model is the following one:

(2) $$\mathrm{PL}(t)=m(f,\;s,\;d_{A},\;d_{B})+w(t)+e()$$

where:

*PL(t)*: propagation loss while transmitting from node A to node B.
*m( )*: propagation loss without random and periodic components; obtained from regression of MMPE data.
*f*: frequency of transmitted acoustic signals (in kHz).
*d_A_*: sender’s depth (in meters).
*d_B_*: receiver’s depth (in meters).
*s*: Euclidean distance between nodes A and B (in meters).
*w(t)*: periodic function to approximate signal loss due to wave movement.
*e()*: signal loss due to random noise or error.

The *m()* function represents the propagation loss provided by the MMPE model. According to the logarithmic nature of the data, a nonlinear regression is the best option to provide an approach to the model based on the coefficients, *An*, supplied by the preliminary model. The proposed expression to calculate this function is the following one:

(3) $$\begin{array}{c} m(f,\;s,\;d_{A},\;d_{B})=\text{log}\left(\left|\frac{{\left(\frac{s}{0.914}\right)}^{A0}\;{\left(d_{A}\right)}^{A9}\;s^{A7}\;{\left({\left(d_{A}-d_{b}\right)}^{2}\right)}^{A10}}{{\left(s*d_{B}\right)}^{10\;A5}}\right|\right)+\\ \left(f^{2}\left(\frac{A1}{1+f^{2}}+\frac{40}{4100+f^{2}}+0.00275\right)+0.003\right)*\left(\frac{s}{914}\right)+A6*d_{B}+A8*s \end{array}$$

The *w()* function approximates the signal loss due to the wave movement. It considers the movement of a particle that will oscillate around its location in a sinusoidal way [13]. That movement is represented as circular oscillations that reduce their radius as the depth of the particle increases. The length of that radius is dependent of the energy of the wave and is related to its height value. The common waves have hundreds of meters of wavelength and have an effect up to 50 m of depth.

For the calculation of the effects of the wave we will consider:

(4) $$w(t)=h(l_{w},\;d_{B},\;t,\;h_{w},\;T_{w})\;\;E(t,\;T_{w})$$

where:

*h()*: scale factor function.
*l_w_*: ocean wave length (meters).
*d_B_*: depth of the receiver node (meters).
*h_w_*: wave height (meters).
*T_w_*: wave period (seconds).
*E()*: function of wave effects in nodes.

This function contains the elements that resemble the node movement, first by calculating the scale factor *h( )* and then the effect of the wave in a particular phase of the wave motion. The calculation of the scale factor is as follows:

(5) $$h\left(l_{w},\;d_{B},\;t,\;h_{w},\;T_{w}\right)=\frac{\left(h_{w}\left(1-\left(\frac{2d_{B}}{l_{w}}\right)\right)\right)}{0.5}*\left|\sin\left(\frac{2\pi \left(\mathrm{mod}\;T_{w}\right)}{T_{w}}\right)\right|$$

The *e()* function represents a random term to explain background noise. As the number of sound sources is large and undetermined, this random noise follows a Gaussian distribution and is modeled to have a maximum of 20 dB at the furthest distance. This function is calculated by the following equation:

(6) $$e()=20\left(\frac{s}{s_{\max}}\right)\;R_{N}$$

where:

*e()*: random noise function
*s*: distance between the sender and receiver (in meters).
*s_max_*: maximum distance (transmission range in meters)
*R_N_*: random number from a Gaussian distribution centered in 0 and with variance 1.

### 2.3. Bellhop (BH)

Bellhop Ray Tracing requires the solution of the ray equations to determine the ray coordinates of the acoustic signal propagation. Amplitude and acoustic pressure requires the solution of the dynamic ray equations which are described in detail in [14]. This tool is integrated with empirical data updated from world databases that measure the Sound Speed Profile (SSP), bathymetry and floor sediment such as the General Bathymetric Chart of the Oceans (GEBCO) and National Oceanic and Atmospheric Administration (NOAA) [15,16]. The ocean wave’s motion is also included to calculate the rays’ trajectories; so taking into account the type of sediments and the sound speed profile (SSP) this propagation model shows a behavior that it is very close to experimental studies for acoustic propagation in underwater environments (more details can be found in [14,17]).

For a system with cylindrical symmetry, the ray equations can be written as:

(7) $$\frac{\mathrm{dr}}{\mathrm{ds}}=c\xi (s)\;\;\;\;,\;\;\;\;\;\frac{d\xi }{\mathrm{ds}}=-\frac{1}{c^{2}}\;\frac{\partial c}{\partial r}$$

$$\frac{\mathrm{dz}}{\mathrm{ds}}=c\zeta (s)\;\;\;\;,\;\;\;\;\;\frac{d\zeta }{\mathrm{ds}}=-\frac{1}{c^{2}}\;\frac{\partial c}{\partial z}$$

where *r(s)* and *z(s)* represent the ray cylindrical coordinates and *s* is the arclength along the ray; the pair *c(s) [ξ(s),* *ζ(s)]* represents the tangent versor along the ray. Initial conditions for *r(s)*, *z(s)*, *ξ(s)* and *ζ(s)* are

(8) $$r(0)=r_{s}\;\;\;\;,\;\;\;\;\;z(0)=z_{s}\;\;\;\;\;,\;\;\;\;\;\xi (0)=\frac{\cos\;\theta_{s}}{c_{s}}\;\;\;,\;\;\;\;\;\zeta (0)=\frac{\sin\;\theta_{s}}{c_{s}}$$

where *θ_s_* represents the launching angle, *(r_s_, z_s_)* is the source position, and *c_s_* is the sound speed at the source position. The coordinates are sufficient to obtain the ray travel time:

(9) $$\tau =\int_{\Gamma }\frac{\text{ds}}{c(s)}$$

which is calculated along the curve *[r(s), z(s)]*.

**Figure 1.**
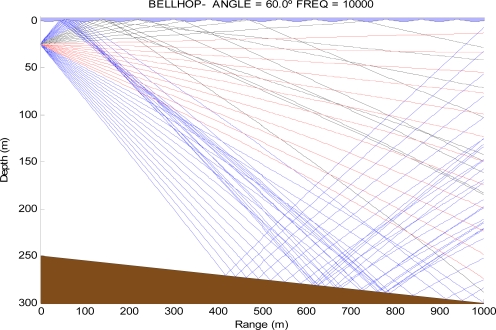
Bellhop ray trace.

Figure 1 shows how the Bellhop tool draws the ray trajectories to calculate the travel of the acoustic signals and thus obtain the attenuation at different points of the scenario.

### 2.4. Bellhop-Based Statistical Prediction Model (BH-SPM)

While the Bellhop model provides an accurate calculation of the acoustic propagation model, network modeling requires near continuous references to propagation delays and signal attenuation values, being computationally infeasible to support this complex model in UWSN simulation frameworks. Thus, it would be of interest to provide an approximation of Bellhop model that supports the time constraints of the network simulation while preserving most of its accuracy.

In [7] we proposed a statistical model based on the Bellhop approach (BH-SPM) that it is able to produce the acoustic signal attenuation map by means of a statistical prediction model integrated in the simulator tool that significantly reduces complexity. BH-SPM model enables computationally-efficient inclusion of fading and multipath effects into a network simulator. Namely, to assess the average system performance, network operation has to be simulated over a large set of channel realizations (e.g., varying surface conditions). Whereas repeated computation of the ray trace for different hops that each of the data packets traverses in a given network may be computationally prohibitive, statistical modeling requires only a single call to the Gaussian random generator for each packet transmission. Thus, the overall simulation time is considerably reduced, allowing a system designer to freely experiment with varying protocols and resource allocation strategies in an efficient manner. The ultimate goal of such computational experiments is to choose the best upper-layer protocol suite and to relate the necessary system resources (power, bandwidth) to the propagation conditions, *i.e*., to the statistical parameters of the transmission loss.

**Figure 2.**
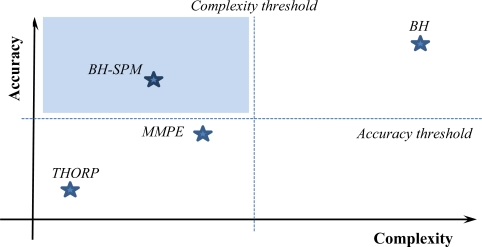
Tradeoff between model propagation accuracy and computational complexity.

In Figure 2, the tradeoff between model complexity and accuracy is shown. In this figure, we also define the thresholds for the desirable accuracy and complexity of the sound propagation model. Thus, the shaded area covers those propagation models with the minimum acceptable model propagation accuracy that leads to get reliable results and, at the same time, low computational complexity levels that allow detailed and scalable network simulations.

**Figure 3.**
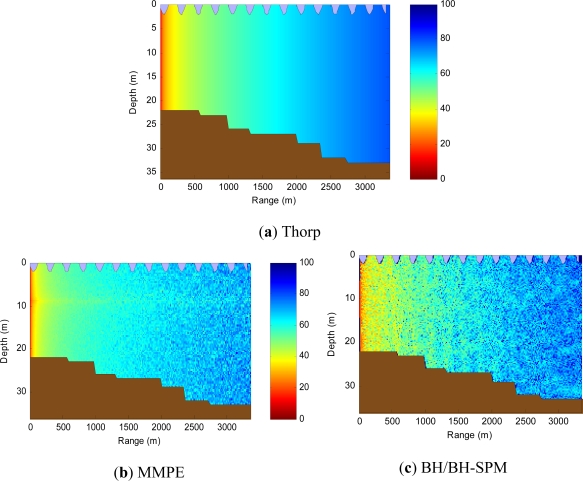
Attenuation of acoustic waves.

In Figure 3 the acoustic attenuation map from the selected propagation models is shown. It was obtained with a specific underwater scenario where a source node, located at 10 m depth, is emitting an acoustic beam of 120 degrees at 10 kHz. The scenario environmental conditions are the same (bathymetry, surface activity, temperature, *etc*.). As it can be shown, the waves (at top) and the underwater floor (at bottom) are represented in blue and brown colors, respectively. We can quickly compare the attenuation values for the different models whereas in (a) Thorp, the simple model only shows signal degradation in accordance with the distance without taking into account neither the scenario depth nor the source radiation pattern, in (b) the MMPE model is able to define a more accurate attenuation map taking into account, depth, distance and ocean wave activity. Finally, in Figure 3(c) the acoustic physics are taken into account by using Bellhop model which introduces the ray reflections/absorptions depending on floor sediment composition, the floor shape and the surface wave’s geometry. In addition, a different sound speed profile is calculated based on the scenario world location and its bathymetry.

Once these models are presented, the next step is to determine if the differences appreciated in Figure 3 may be transferred to the upper network layers in such a way that performance of higher layer protocols is affected in a significant way. If so, then it would be necessary to consider the use of complex propagation models that represent as accurate as possible the underwater acoustic propagation in order to obtain simulation results close to the ones obtained in experimental test-beds.

## 3. Higher-Layer Protocols: MAC & Routing

After reviewing and analyzing several acoustic propagation models, in this section we will introduce several higher network layer protocols that we will use later in our experiments. Most of the higher protocols presented below were proposed for terrestrial network technologies, and their network performance is well-known. However, as stated in the introduction, UWSNs exhibit several architectural differences with respect to the terrestrial ones. In particular, the acoustic signal propagation delay is several orders of magnitude higher than RF signals, resulting in large signal propagation *versus* packet transmission time ratios, something which it is not desirable for obtaining high utilization levels of network resources, and as a consequence limiting the overall network performance. So, the behavior of higher level protocols may seriously change when they are employed in UWSN scenarios.

As with acoustic propagation models, the selected protocols for the evaluation include: (a) several MAC layer protocols: from simple proposals, like ALOHA that only cares of sending the packet message, until more complex protocols like DACAP that include carrier sense and collision avoidance by means of at least three-way handshake exchanges; and (b) a couple of routing protocols: a simple static routing and a cross-layer routing proposal FBR. We will test all of them to determine how the scenario environmental changes impact on their performance in terms of throughput and delay.

### 3.1. ALOHA

The ALOHA protocol [18] is the simplest MAC protocol since it does not care about channel status or packet delivery success. So, it quickly reaches the network saturation point producing a huge number of collisions. This MAC approach is avoided in other network technologies due its lack of ability to proper order the access to a shared medium, but it does not requires handshaking exchange.

### 3.2. CSMA

The Carrier Sense Multiple Access (CSMA) [19] is an evolution of ALOHA that includes a channel sensing mechanism, so before sending a data packet, the CSMA protocol checks if the shared channel is free. If not, it defers data packet transmission until the channel is freed. This protocol reduces considerably the channel collisions when compared with the ALOHA protocol, without requiring extra signaling.

### 3.3. CSMA/CA

The Carrier Sense Multiple Access with Collision Avoidance (CSMA/CA) incorporates a handshaking process to establish the communication channel between two nodes. It uses request to send (RTS) and clear to send (CTS) control packets to create a tunnel free of collisions at both communication ends. After acquiring the channel, the data packet is sent to the destination node. Finally, the sender waits for an acknowledgment control packet (ACK) that will indicate the successful reception of data packet. If no ACK is received, then a contention mechanism, typically based on a back-off scheme, randomly delays the packet retransmission. Also, a maximum number of consecutive retransmission attempts is defined. If this maximum is reached, then the packet is discarded.

### 3.4. DACAP

Distance Aware Collision Avoidance Protocol (DACAP) [20] is a handshaking protocol designed for *Ad Hoc* Underwater Acoustic Sensor Networks. The protocol includes a power aware behavior that that it is intended to reduce power consumption by avoiding/reducing collisions and at the same time achieving good network throughput. It also minimizes the handshake time by using the tolerance to interference of receiver node, especially when receiver is close to the reception range limits. The network nodes do not need to be synchronized and it supports node mobility (dynamic scenarios). The throughput achieved by DACAP is several times higher than the one achieved with Slotted FAMA [18], while offering similar protection from collisions.

The improvement introduced by DACAP towards the traditional CSMA/CA mechanism lies on the behavior of the receiving node when is waiting for a data packet. If it overhears a control message coming from other node, it will send a warning packet (WAR) to this node in order to let it know that there is already a transmission in process. Moreover, after receiving the CTS control packet, the sender node defers the transmission data packet for a defined delay time. The transmission attempt is aborted if by any chance the sender node receives another control or warning message. The delay times are determined according to the distance between the nodes involved in the transmission, that can be solve during the handshake by measuring the roundtrip time. Even though when the receiver node sends a warning message, it has no feedback that lets it know if the interfering node cancels its transmission. That is the reason why the receiver keeps listening to the channel after sending the warning message, and thus the defer state is set to a minimum delay time between the CTS and the DATA so that it avoids a collision.

### 3.5. Static Routing

This kind of routing protocol identifies a simple approach at routing layer, where the path between two network nodes is always the same in those networks without node mobility (every node is fixed at a specific location). Although there are several routing protocols that fit into this category, the geographical-based routing protocols are very popular in Wireless Sensor Networks deployments, where all network nodes are equipped with a localization system that determine their global position. So, when a source node has to deliver a packet to a destination node located several hops away, it would forward the packet to the neighbor node that is closer to the destination. Assuming that network topology is static (no mobility pattern), the path provided by a static routing protocol to reach a specific network node will be always the same.

### 3.6. FBR

The Focus Beam Routing (FBR) [21] protocol is based on node location capabilities, and is able to find the path between two nodes in a random deployed network if each node knows its position and the position of the destination (gateway) node. Assuming a communication between nodes A and B, node A will send an RTS multicast packet to all the reachable neighbors. This packet will include the information of the source (node A) and final destination (node B). This request is a short control packet that contains the location of both the source node (A) and the final destination (B) node. Packet collisions can happen but always will involve short packets as the link is safe for data packets which have no risk of collisions. Although the chances of collision are small, if the source node detects a collision, it will receive signal but it will not be able to decode the information of the incoming data frame, it will resend the RTS once again.

## 4. Propagation Model Evaluation

In this section, we will analyze the behavior of different propagation models when simulating an underwater wireless sensor network deployed in a specific network scenario location. We will study both performance results and sensibility under network scenario parameters. For that purpose we will describe the characteristics and parameters associated to the network scenario, the MAC protocol we will employ, and the traffic load characterization.

### 4.1. Scenario Deployment

**Figure 4.**
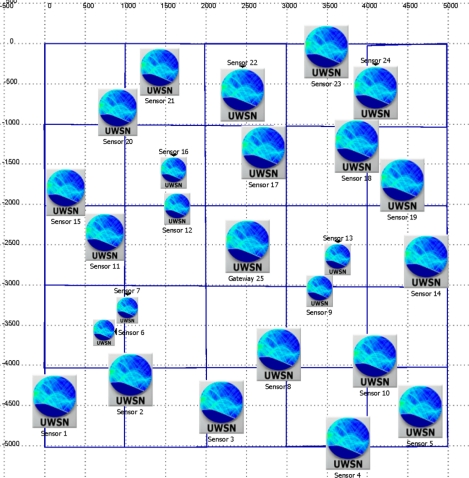
Network deployment 2D.

The network scenario deployment is shown at Figure 4 (surface view). The volume size is defined as a cube of 5,000 × 5,000 × 50 m (Length × Width × Depth); the covered area is divided in cells of 1,000 × 1,000 m. The gateway (sink node) is always placed in the middle cell at a fixed depth of 10 m. Then we put one node per cell in a random position inside the cell, as well as random depth (this parameter will be bounded by the scenario bathymetry). Once all the nodes are deployed the connectivity of the network is checked by guaranteeing that every node has a path to the gateway (one-hop or multi-hop paths) and that there are not isolated subnetworks or nodes. Using the same area and cell size, ten different random scenarios have been built and validated for the simulation test.

**Figure 5.**
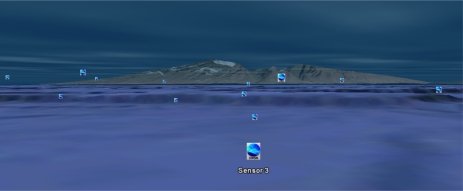
Network deployment 3D (Google Earth™).

In Figure 5, a 3D representation of the network scenario is shown. It is located at coordinates 39°48′13.14″N and 0°4′34.53″W (Valencia, Spain). This view let us appreciate the different depths of the nodes, close to the surface, middle depth and at the bottom of the scenario. We have fixed the wave activity with waves of 2 m height and 80 m length. The network scenario floor is composed of gravel. All of the scenario and environmental parameters were taken from National Geophysical Data Center databases [22,23] related to the specific global coordinates of our network. This example could represent a typical network scenario of shallow waters with a low altimetry shape where the bottom relief is deeper as it goes farther from the coast.

In Table 1 the main parameters used in the simulations are shown.

With respect to network traffic load, we proposed a constant bitrate approach where every sensor node generates fixed length data packets at a generation rate defined with an exponential distribution. All sensor nodes in the network will send packets towards gateway node, so we will obtain a hot-spot traffic pattern, where all the packets are delivered to the same destination.

In this section we will consider a One-Hop (OH) network topology, where all network nodes are able to reach the gateway in one hop. The power transmission is set constant in all nodes and it is calculated as the energy required by the farthest nodes (the ones in the perimeter) to reach the gateway, considering Thorp’s attenuation model. That means that in Thorp simulation these nodes will always reach the gateway but in both MMPE and Bellhop reachability it is not guaranteed, mainly due to the more realistic assumptions about the acoustic propagation. So, the time-varying acoustic signal attenuation may produce packet loses due to the lack of signal strength at gateway, which is supposed to have an impact on the network performance.

**Figure 6.**
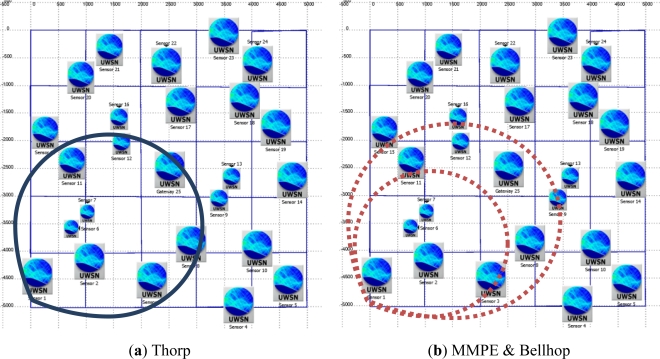
Reachability of gateway from Node #1 (bottom leftmost).

In Figure 6(a) the transmission range of node #1 is fixed (are covered by the circle) during all the simulation, indicating the set of nodes that always receive the transmissions from node #1 (in particular the gateway node) using Thorp propagation model. However, in Figure 6(b), MMPE and Bellhop models define the transmission range with two dashed circles. The smaller circle represents the nodes that always receive node #1 transmissions; meanwhile the rest of nodes included in the bigger circle (those that are out of the smaller one) may receive the transmissions with a certain probability defined by the propagation model.

### 4.2. Evaluation Results

In this section we are going to evaluate the different propagation models proposed in Section 3, assuming we have a CSMA/CA MAC protocol and the scenario and simulation parameters defined above.

The simulation framework is based on OPNET, MATLAB and Bellhop ray tracing tool, and uses information related to underwater scenario characteristics like bathymetry, salinity, and seafloor composition, found at real worldwide locations which it is downloaded from NOAA and GEBCO worldwide ocean databases. This information is combined with the OPNET network scenario module in order to create the corresponding environmental files. With these files OPNET connects to MATLAB through its interface and runs Bellhop obtaining the result files. A fully explanation of the simulator framework can be found in [24].

The performance metrics we will show are:

1. **Goodput**, defined as the throughput found at the application layer (note that in top of MAC layer we have no other network layers, only the application), so only data packets that successfully arrive to the gateway node are taken into account. This also means that control packets like RTS, CTS, and ACK are not considered in the computation of goodput.
2. **Average Packet Delay**, defined as the average delay incurred by a packet to reach its destination. This delay is calculated from the time when MAC layer gets the packet at source node to start delivery until the instant when this packet is correctly received at gateway node.

**Figure 7.**
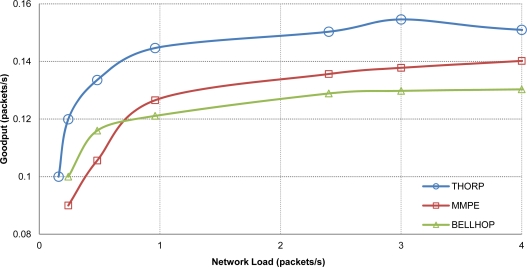
Average Goodput with different acoustic propagation models.

In Figure 7, we can see the average goodput from 10 random scenarios (as defined in previous subsection) is shown. As it can be observed, the results appear to follow the same pattern with clearly different goodput values depending on the propagation model used. The Thorp propagation model gets the best performance, MMPE is estimable worse and finally Bellhop is the one with the worst behavior. This behavior agrees with the prediction stated before as the connection links between the nodes that are farther from gateway suffer the consequences of using more accurate propagation models like MMPE and Bellhop. In other words, Thorp model always provides link reachability to network nodes during the simulation; however MMPE loses communication due to the wave effect and this leads to reduced goodput performance. This behavior is even more pronounced with Bellhop model where signal attenuation is calculated in a more accurate way, resulting in a higher number of dropped packets during the n-way handshaking process of CSMA/CA protocol.

**Figure 8.**
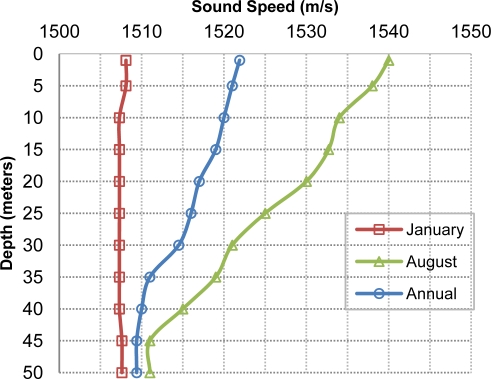
Valencia’s (scenario location) annual average sound speed as a function of node depth.

On the other hand we measure the average packet delay which will strongly depend on the channel propagation delay. So, the propagation delay (*T_prop_*) depends on the distance (*d*) between sensor and gateway nodes, the specified inter frame delay (*SIF*), and the sound speed propagation (*T_ssp_*) that may change with node depth and water temperature, as shown in Figure 8 obtained through databases [22,23].

In expression (10) we define the delay experienced by a packet delivery in one-hop transmission without network contention/interference, taking into account the CSMA/CA protocol handshake and the distance and sound speed parameters.

(10) $$\begin{array}{c} T_{\mathrm{prop}}=d/T_{\mathrm{ssp}},\;T_{\mathrm{pkt}}=\mathrm{packet\_size}/\mathrm{data\_rate},\;\mathrm{SIF}=\mathrm{Inter\_Frame\_Delay}\\ \mathrm{Delay}=T_{\mathrm{prop}}\;(\mathrm{RTS})+T_{\mathrm{pkt}}\;(\mathrm{RTS})+\mathrm{SIF}+T_{\mathrm{prop}}\;(\mathrm{CTS})+T_{\mathrm{pkt}}\;(\mathrm{CTS})+\mathrm{SIF}+T_{\mathrm{prop}}\;(\mathrm{DATA})+T_{\mathrm{pkt}}\;(\mathrm{DATA}) \end{array}$$

So the experienced delay of packet sent by a sensor located 1,500 m away from gateway node and with 10 m depth would be:

(11) $$\begin{array}{c} T_{\mathrm{prop}}\;(\mathrm{RTS})=T_{\mathrm{prop}}\;(\mathrm{CTS})=T_{\mathrm{prop}}\;(\mathrm{DATA})=1,500/1,520=0.9868\;s\\ T_{\mathrm{pkt}}\;(\mathrm{RTS})=T_{\mathrm{pkt}}\;(\mathrm{CTS})=24/5,000=0.0048\;s\\ T_{\mathrm{pkt}}\;(\mathrm{DATA})=1,024/5,000=0.2048\;s\\ \mathrm{SIF}=0.02048\;s\\ \mathrm{Delay}=3*0.9868+2*0.0048+0.2048+2*0.02048=3.21576\;s \end{array}$$

**Figure 9.**
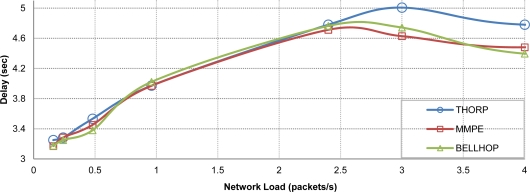
Average packet delay with different acoustic propagation models.

The results shown in Figure 9 reveal almost the same delay for all propagation models until the network enters in a saturation state, where MMPE and Bellhop seem to produce better results. At first sight this may suggest a lack of coherence, but if we take a close look at the distribution of packets received in the gateway from the different source nodes, we will appreciate that with MMPE and Bellhop, the gateway receives less packets from the farther nodes as they are more affected by the attenuation variability introduced by these propagation models, as shown before in Figure 6, so, this is the main cause of the lower overall packet delay with the use of more accurate acoustic propagation models, since the average packet delay decreases.

In the early first tests, it is clear that the propagation model is an important issue to take into account but now we go a bit further by changing the environmental parameters of the network scenario in order to assess their influence. For that purpose we will use one of the scenarios used before, fixing the network load at 2 packets/s to the point just before network saturation, and introducing two different months of the year (January and August) with different ocean average temperatures plus six different levels of wave heights (varying surface conditions) from 1 to 11 m height, the rest of network and environmental parameters remain the same as in Table 1.

**Figure 10.**
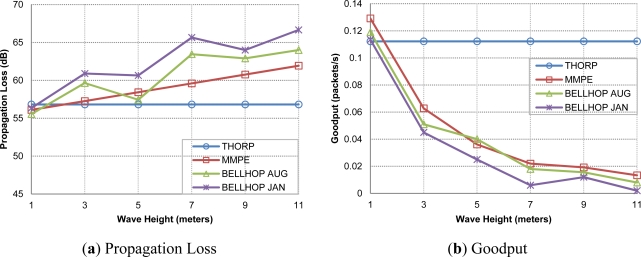
Propagation loss (**a**) and goodput (**b**) values varying physical scenario parameters.

In Figure 10(a) the acoustic attenuation found between two network nodes, sensor 1 and gateway, is shown. As expected, Thorp’s results remain constant since its equation does not include the effect of the physical parameters. Meanwhile, MMPE and Bellhop propagation models significantly reduce the obtained goodput as the wave height increases, *i.e*., they suffer from the wave motion effect. Also, neither Thorp nor MMPE are affected by the change of season whereas Bellhop shows different results for the months selected, we can appreciate worse performance in January than in August due to the different propagation conditions derived from the average ocean temperature. The average delay results including variable physical parameters are not included here as they are almost the same behavior than in Figure 9.

So, summarizing this section, we can observe that in addition to having a detailed propagation model, different environmental conditions have a great impact in network performance. This leads us to seriously consider both: (a) an accurate acoustic propagation model, and (b) environmental and scenario parameters to obtain reliable simulation results that efficiently predict the real behavior of the sound propagation in a particular network scenario.

## 5. Higher Layer Protocol Evaluation

In the previous section we have reached interesting conclusions about the influence of the propagation models, so in this section we are going to evaluate their impact on different higher layer protocols, MAC and routing protocols, using an accurate propagation model like the one defined by Bellhop and taking into account several physical parameters related to the network scenario and environmental conditions.

In the following evaluation we will reuse the same network scenarios define in previous sections but in two different operational modes: One-Hop (OH) and Multi-Hop (MH). In the first one, OH, all network nodes are able to directly reach gateway node (packet destination); whereas the later mode, MH, some network nodes require relaying their packets through other nodes to reach the gateway.

**Figure 11.**
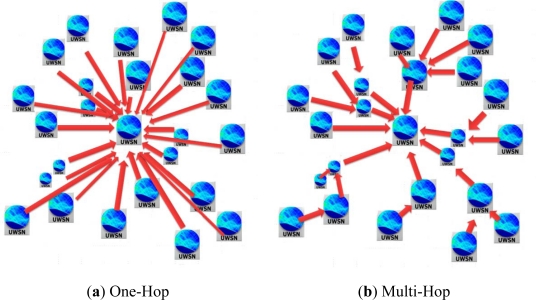
Network operational modes.

The difference between OH and MH modes is focused in the allocated transmission power level to network nodes, which define their coverage area, so in OH network scenarios we adjust transmission power level to reach gateway node from farthest nodes (the same as in previous section simulation experiments). However, for MH network scenarios we will reduce the power transmission of nodes in such a way that they will be able to reach only the nodes of the adjacent cells. In MH network scenarios, a routing protocol is required to let the packets travel across the network towards their destination (gateway node). By default, in MH network scenarios we define a static routing protocol. In Figure 11 we can see the connections between nodes in both operational modes.

### 5.1. MAC Protocols

In the first test we choose two MAC protocols: CSMA and CSMA/CA. Although they seem to be very similar approaches, CSMA it is a simple version with no signaling to handle a packet transmission, meanwhile CSMA/CA is a 4-way handshake protocol as defined in Section 3.

Our purpose is to analyze how these MAC protocols tackle a network deployment with different power transmission policies, clearing up where it is worth to focus the efforts in terms of power consumption, throughput, packet delay, *etc*. The simulation parameters are the same as in Table 1 increasing the global load up to 12 packets/s.

**Figure 12.**
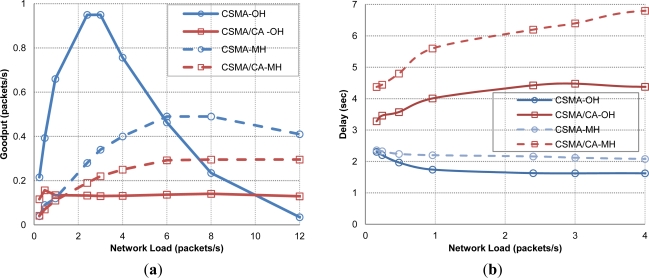
Goodput (**a**) and delay (**b**) of selected MAC protocols in OH and MH modes.

In Figure 12(a), we can see that CSMA-OH soon reaches its highest performance, and after saturation point its goodput performance degrades very quickly reaching a near network starvation state. However, CSMA-MH follows the same pattern with a smoother curve. This behavior in OH scenario can be easily explained because at lower loads the gateway receives more or less the same number of packets from all network nodes, while in MH scenarios the effect of hot-spot traffic pattern leads to unbalance this behavior and as a consequence reduce network load in the gateway neighborhood.

In turn the CSMA/CA evolution is similar in both strategies, quickly reaching its best performance and keeping it despite the increasing load; having a better overall result in the MH strategy due to the same reasons commented before. It is important to remark that at higher network loads, in all cases but especially in MH, sensors that are closer to the gateway have more chances to achieve successful data packet transmissions than farther nodes (no fair resource sharing due to hot-spot traffic pattern and the inherent large propagation delay).

From these results we can state that the MH strategy has an overall better performance, being at the same time more energy efficient since it is able to reduce energy demands to half of the ones required by OH. Also, it was observed that those nodes located at the scenario surroundings will have less probability to successfully deliver packets to gateway, so this issue opens the way to define routing protocols that will balance the overall packet delivery rate between all the sensor nodes with independence of their location.

Now, we take a look at the delay behavior shown at Figure 12(b). As stated in Equation (10) the CSMA/CA delay is the result of the acoustic propagation time and the transmission time of the different packets involved in the handshaking, meanwhile in CSMA we only send DATA packets so it is expected that it gets smaller delays. As shown in Figure 12(b) CSMA delays are slightly higher in MH than in OH. This result obeys to the fact that the signal propagation delay between a source node and the gateway is typically smaller than the sum of propagation delays of the paths followed to reach gateway node, plus the time required to send at least *n* data packets (where *n* is the number of hops to reach gateway) instead of one:

(12) $$\begin{array}{c} \mathrm{OH-Delay}=T_{\mathrm{prop}}\;(O)+T_{\mathrm{pkt}}\;(\mathrm{DATA})\\ \mathrm{MH-Delay}=T_{\mathrm{prop}}\;(M1)+T_{\mathrm{prop}}\;(M2)+2*T_{\mathrm{pkt}}\;(\mathrm{DATA})\\ T_{\mathrm{prop}}\;(O)\leq T_{\mathrm{prop}}\;(M1)+T_{\mathrm{prop}}\;(M2) \end{array}$$

**Figure 13.**
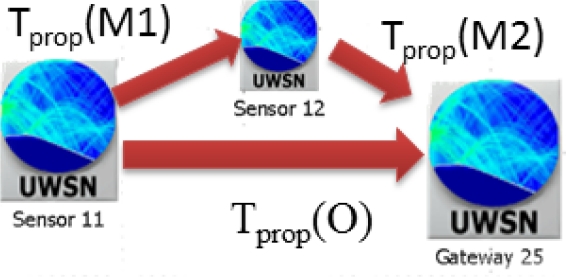
Signal propagation times: OH *vs*. MH.

In Figure 13, we show an example involving two network nodes and the gateway. The propagation time from sensor 11 to gateway use to be smaller than the propagation from sensor 11 to 12 plus the propagation from sensor 12 to gateway. In the event where OH and MH strategies suffer the same propagation delay, MH mode would require two data transmission cycles, so the overall packet delay is always longer than in the OH strategy. This fact has a greater influence in ann-way handshaking protocol, like CSMA/CA, where for each data packet transmission (each hop) *n* packet propagation delays are required, increasing a lot the overall packet delay.

The CSMA/CA protocol shows a more stable behavior in terms of goodput performance in OH network scenarios, but environmental conditions and the influence of propagation time in the overall packet delay dramatically affect handshaking protocols. On the other hand, the CSMA protocol maintains the average packet delay low and stable in both OH and MH strategies, since no handshaking is performed to complete one packet delivery. In Figure 12(b), CSMA protocols exhibit higher average packet delay at very low network loads, decreasing as network loads increase. This behavior is due to the hot-spot traffic pattern, since as network load increases the nodes closer to the gateway are the ones with higher delivery rates and, at the same time, lower packet delay (signal propagation delay), resulting in a reduction of the average packet delay.

**Figure 14.**
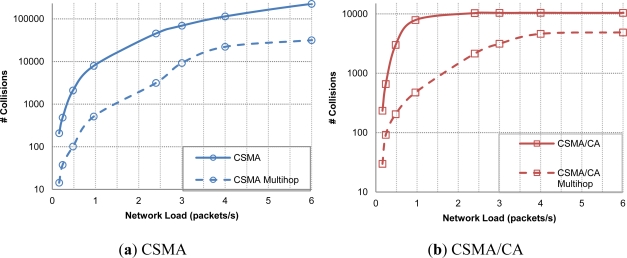
Collisions one hop *vs*. Multihop.

Finally, another gauge to measure the power consumption in the network is packet collision statistic. In Figure 14, we show CSMA and CSMA/CA protocols with both OH and MH network configurations. As expected, CSMA shows a much higher number of collisions, leading to an increasing number of packets lost, increasing the overall wasted energy. However, CSMA/CA shows better performance arriving to a constant number of collisions just after the network reached saturation. In both cases, the number of collisions is highly reduced with the MH approach.

### 5.2. Routing Protocols

In this subsection we perform a simple simulation experiment with a particular MAC protocol in combination with different routing policies, in order to assess their behaviour under different scenario environmental conditions. We propose the DACAP MAC protocol since it defines some crosslayer support for routing protocols, and we want to quantify the benefits of crosslayer approaches when the scenario environmental conditions change. So we will test the behaviour of DACAP MAC protocol with two routing protocols, a static routing protocol (always supplies the neighbour with the nearest node to gateway) and the FBR routing protocol. Also we will include in our experiments two different propagation models, Thorp and Bellhop. For the simulation parameter we will use the ones in Table 1 except for the propagation models, we only use Thorp and Bellhop, the global network load is fixed to 3 packets/s, and we define an MH scenario configuration.

**Figure 15.**
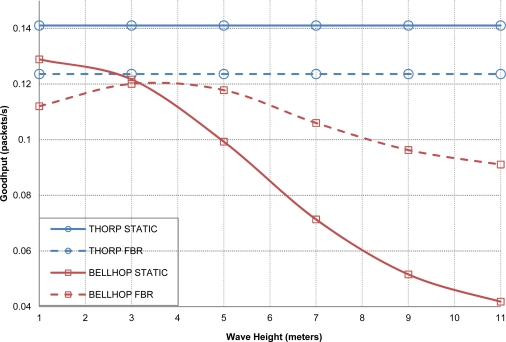
Goodput results with DACAP + Routing using two different propagation models.

Figure 15 depicts and interesting behavior of DACAP protocol in terms of goodput performance. In addition there is a clear indication that also, at the routing layers, the environmental conditions of the network scenario may considerably impact in the results of network performance.

As also shown in previous results, the Thorp propagation model does not take into account environmental conditions, so it plots a constant goodput value. As, expected the static routing protocol gets better goodput results because FBR has an extra waiting time in order to accept more than one CTS, but as every node has always the same reachable nodes in its neighborhood it always choses the same node to reach to the gateway, that is the reason why using static routing in ideal conditions is a better option. If we use Bellhop the static alternative loses performance as the attenuation grows due to the physical changes, meanwhile FBR performance is not so affected in worse conditions as it can dynamically change the routing paths when a connection link is lost.

## 6. Conclusions

One of the main difficulties to compare and validate the performance of different UWSN proposals is the lack of a common standard to model the acoustic propagation in the underwater environment. In this paper we analyzed several underwater acoustic propagation models from a simple approach to more detailed and accurate models, in order to study whether differences between then may seriously impact in the performance evaluation of higher layer protocols. As a first conclusion we found that accurate and low-complexity propagation models are required for network simulation in order to obtain reliable results attained to the specific scenario and environmental parameters.

Also we perform several simulation experiments to determine the sensibility of higher layer protocols (MAC and routing protocols) to propagation models and scenario environmental parameters. From the obtained results, we conclude that: (a) n-way handshake protocols, like CSMA/CA or DACAP suffer from high packet delays, but they show better behavior in terms of goodput and energy consumption; and (b) crosslayer approaches between routing and MAC layers are required to improve network performance, so it is highly recommended to allow routing protocols to get appropriate feedback from MAC layer about network and environmental conditions found at physical layer, since in UWSNs we showed the impact of physical layer modeling on network performance.

The importance of not only choosing a realistic propagation model but also defining with precision the environment, starting with the geographic position and the parameters that we can obtain from it to the physical environment conditions like the season of the year or the ocean wave motion has been settled, so when designing network architectures for UWSNs, the role of physical layer should be seriously taken into account to be in a position to assert that simulation results will be close to the ones obtained in real network scenarios.

## Figures and Tables

**Table 1.** Simulation and network scenario parameters.

**Parameter**	**Value**
Propagation Models	THORP, MMPE, BELLHOP
# Sensors	24
# Gateways	1
Month	Annual Average
Wave Height (m)	2
Wave Length (m)	80
Frequency (kHz)	10
Scenario Depth (m)	50
Global Load (packets/s)	0.16 to 4
Data Packet Size (bits)	1,024
Control Packet Size (bits)	24
Bandwidth (kbps)	5
# Scenarios	10
Simulation Time (s)	3,600
